# Systematic validation of predicted microRNAs for cyclin D1

**DOI:** 10.1186/1471-2407-9-194

**Published:** 2009-06-18

**Authors:** Qiong Jiang, Ming-Guang Feng, Yin-Yuan Mo

**Affiliations:** 1 Department of Medical Microbiology, Immunology and Cell Biology, Southern Illinois University School of Medicine, Springfield, IL, USA; 2 Institute of Microbiology, College of Life Sciences, Zhejiang University, Hangzhou, PR China

## Abstract

**Background:**

MicroRNAs are the endogenous small non-coding RNA molecules capable of silencing protein coding genes at the posttranscriptional level. Based on computer-aided predictions, a single microRNA could have over a hundred of targets. On the other hand, a single protein-coding gene could be targeted by many potential microRNAs. However, only a relatively small number of these predicted microRNA/mRNA interactions are experimentally validated, and no systematic validation has been carried out using a reporter system.

**Methods:**

In this study, we used luciferease reporter assays to validate microRNAs that can silence cyclin D1 (CCND1) because CCND1 is a well known proto-oncogene implicated in a variety of types of cancers. We chose miRanda http://www.microRNA.org as a primary prediction method. We then cloned 51 of 58 predicted microRNA precursors into pCDH-CMV-MCS-EF1-copGFP and tested for their effect on the luciferase reporter carrying the 3'-untranslated region (UTR) of CCND1 gene.

**Results:**

Real-time PCR revealed the 45 of 51 cloned microRNA precursors expressed a relatively high level of the exogenous microRNAs which were used in our validation experiments. By an arbitrary cutoff of 35% reduction, we identified 7 microRNAs that were able to suppress Luc-CCND1-UTR activity. Among them, 4 of them were previously validated targets and the rest 3 microRNAs were validated to be positive in this study. Of interest, we found that miR-503 not only suppressed the luciferase activity, but also suppressed the endogenous CCND1 both at protein and mRNA levels. Furthermore, we showed that miR-503 was able to reduce S phase cell populations and caused cell growth inhibition, suggesting that miR-503 may be a putative tumor suppressor.

**Conclusion:**

This study provides a more comprehensive picture of microRNA/CCND1 interactions and it further demonstrates the importance of experimental target validation.

## Background

Accumulating evidence indicates that microRNAs could play a fundamental role in regulation of diverse cellular pathways including differentiation, proliferation and apoptosis [1,2]. Thus, deregulation of microRNAs affects normal cell growth and development, leading to a variety of disorders including human malignancies, which have been demonstrated in both in vitro and in vivo systems [1,3-6]. Importantly, tumor cells often express a unique set of microRNAs (or microRNA signature). For example, the overall microRNA expression profile of normal tissues is distinct from that of tumor tissues [7] and moreover, microRNA signatures are able to accurately identify cancer tissue origin [8]. MicroRNAs can function as tumor suppressors or oncogenes depending on whether they specifically target oncogenes or tumor suppressor genes [9-11]. For instance, tumor suppressive microRNAs such as let-7, miR-15 and miR-16 are downregulated or deleted in lung cancer and leukemia [11-13]. In contrast, oncogenic microRNAs, such as miR-21 and miR-155, are overexpressed in tumors or tumor cell lines [7,14,15].

Since microRNAs are capable of silencing gene expression by binding to the 3'-untranslated region (3'-UTR) with partial sequence homology, a single microRNA usually has multiple targets [16]. Thus, microRNAs could regulate a large fraction of protein-coding genes. As high as 30% of all genes could be microRNA targets [17,18]. In essence, microRNAs can be considered to be modulators of gene regulators and they can cooperate with transcription factors. Together, microRNAs and transcription factors determine gene expression patterns in the cell [19].

Despite the tremendous progress made in the past several years in identification of microRNAs and their role in diverse cellular functions, the mechanism of microRNA-mediated tumorigenesis is still poorly understood presumably because we do not have a clear picture as to how microRNAs silence their target genes. Given the nature of microRNA targeting, a single protein coding gene (mRNA) can be targeted by multiple microRNAs. For example, CCND1 can be potentially targeted by as many as several dozens of microRNAs depending on which prediction method is used. However, it is not clear how many microRNAs actually can target CCND1. Since CCND1 is a key gene controlling cell cycle and it is frequently overexpressed in cancer cells, contributing to cell proliferation and migration [20], we thought that CCND1 would be an idea gene for microRNA target validation because a few microRNAs have been previously validated, such as miR-17/20 [21], miR-16 family [22,23], and let-7 [24]; and a relatively large number of microRNAs are predicted to target CCND1.

Therefore, we attempted to validate these predicted microRNAs in a biological reporter system. We found 7 microRNAs are positive to CCND1. In addition to previously validated microRNAs, we found that miR-503 can cause cell cycle arrest and growth inhibition through in part silencing CCND1. To our knowledge, this is the first systematic target validation effort using the biological reporter system and thus, it is expected to provide more comprehensive view of how microRNAs regulate this important gene.

## Methods

### Cell Culture and Reagents

Human head and neck carcinoma cell line UMSCC10B has been described previously [25] and was grown in DMEM (Cambrex, Walkersville, MD) supplemented with 10% fetal bovine serum (Sigma-Aldrich, St. Louis, Mo), 2 mM-glutamine, 100 units of penicillin/ml and 100 μg of streptomycin/ml (Cambrex). The cultivation of immortalized kidney epithelial cells 293T (ATCC) has been previously described [26]. All cells were incubated at 37°C in a humidified chamber supplemented with 5% CO_2_.

Scrambled LNA (locked nucleic acid) oligonucleotide and anti-*miR-503 *LNA oligonucleotide were purchased from IDT (Coralville, IA). Primary anti-CCND1 antibody (Epitomics, Burlingame, CA) and anti-β-actin antibody (Sigma. St. Louis, Mo) were used in western blot. Secondary antibodies conjugated with IRDye 800CW were purchased from LI-COR Biosciences (Lincoln, NE). PCR primers were purchased from Sigma-Genosys (Woodland, TX) or IDT.

### Plasmid constructs

To construct pre-microRNA expression vectors, we first amplified ~0.5 kb DNA fragment covering a pre-microRNA, using genomic DNA from a healthy blood donor as a template. PCR reactions were performed using the high fidelity Phusion enzyme (New England Biolabs Ipswich, MA) and corresponding specific primers as listed in Table 1. The amplified fragment was first cloned into a PCR cloning vector (pCR8) and subsequently cloned a lentiviral vector (pCDH-CMV-MCS-EF1-copGFP from System Biosciences, Mountain View, CA) at EcoR1 and Not1 sites. Where there was an internal EcoR1 or Not1 site, partial digestions were performed to obtain the DNA fragment carrying the pre-microRNA. Expression of the mature microRNAs was verified by QuantiMir kit (System Biosciences) or TaqMan real-time PCR kit (Applied Biosystems) [27,28].

**Table 1:** Primers used to clone microRNA precursors in this study

MicroRNA	5' primer	3' primer
miR-19a	GCATCTACTGCAGTGAAGGC	GCGGCCGCAGTTTGATTGGGCGACAGG

miR-491	AGATGGCTTTCTGGGTAGCC	GCGGCCGCTAACAGTAACACGTATGGGC

miR-296	CCTCCTTGGAGCTGAGATGG	GCGGCCGCGTGGGAGGTAGGTAAAACTC

miR-532	CATCTGTTCTCGTATGTGTG	GCGGCCGCACGTAAGAGAGGGTTTACC

miR-188	CTTCCCTCTCCAGTGCATAG	GCGGCCGCTCCTGCAGGATCCATGTAAG

miR-579	CAGTGACAGAGTTGTGTAAC	GCGGCCGCGTGGAAACAAGTTGCATGTC

miR-501	AGACCCTCAAGTGTGATCTG	GCGGCCGCAGTATATGCAGGGGCAATG

miR-548c	TCAGGGTTTCACCATGTTGG	GCGGCCGCACAGAGCAAGACTCCATCTC

miR-219-1	CGTTTACTCTAGAGTCCTCG	GCGGCCGCACAGAGGGCTCGTGAAAAGG

miR-340	TGGCTACCTGTTCAGCTTTC	GCGGCCGCAGCAGTTTACCAGAATACCG

miR-519b	CTGGACACAAAACTCAGGAG	GCGGCCGCGTCTGGGTCACAGAGCAAG

miR-155	TGCCTAAAGGTAACAATGTC	GCGGCCGCGGTTGAACATCCCAGTGAC

miR-519e	GGTATGTGTGGTAGGCTTTG	GCGGCCGCAACTAGCCTAAGAGGTCTAG

miR-515-1	TACATGCCACCACAGGCGGC	GCGGCCGCCTGGGCCACAGAGAGAGAC

miR-139	GATCGTGCAGGCAACTCTTG	GCGGCCGCTGGATAACAAGGCAGTATGG

miR-494	TTCCCGGGCAACCTCTTTTC	GCGGCCGCTCCTTCAACCACAGAAGCAC

miR-203	CGTCTAAGGCGTCCGGTACG	GCGGCCGCTTCCCACAGCACAGCCCGGC

miR-142	GATGGGGTGGAGCCTTTAGG	GCGGCCGCAACCAGGAAGGGCAGGAAAG

miR-450-1	TGATTATCTCTGAGTTGTGG	GCGGCCGCAAAATGATCCCAATACAC

miR-193a	AGTTTCTCGGCGCATAACTC	GCGGCCGCTATTTCTCCAGCGAAGTG

miR-193b	GTTGTCCGGGAAGCTTTTAG	GCGGCCGCCCTCGAAGGGACCTTTTAG

miR-487b	TGGTCTGGGTCCCTGCTTCC	GCGGCCGCAGTCATAAAACCACCACCAC

miR-487a	CAGTCTCATGAGGGAGTGCC	GCGGCCGCTCTCACTGGTCAACTTCCC

miR-301	AGGGCTTGTTTTACATGGCG	GCGGCCGCCAGACGTGTTTCATAATGC

miR-130a	CTGCTTTAGTGGGTCCTGTC	GCGGCCGCTAACGGAGGCAGTGTCTATC

miR-130b	GTCAAAGCATCTGGGACCAG	GCGGCCGCACCTGATCCTCTGATGGAG

miR-148a	CTTTGCTGTGACATTGCGAC	GCGGCCGCTTTGGGGGGATTTTGTTCCC

miR-503	CTCGTGGGGAAGGTAGAAGG	GCGGCCGCAAGATAGGTTTTCTTCGTTG

miR-152	AGCAGCCAACTCAGAACTCG	GCGGCCGCAAGGTCCACAGCTGGTTCTG

miR-95	CCTCCTGAGGAATCCAGGCG	GCGGCCGCGATCGGGCCCTTGCGAAGCC

miR-550	GAGCCTTCTAACCACAACCC	GCGGCCGCATAAAGCAGTTCCTACACTC

miR-545	TCATGCCAGTGTCTAGTTCC	GCGGCCGCCACCAAGAAAGCAAGTGTC

miR-424	ACCCACTACGTTGTTCCAAG	GCGGCCGCAGAGCACCGATCGCTCACTG

miR-15a	AAAGGTGTACTGCAAGGAAC	GCGGCCGCACTGCTGACATTGCTATC

miR-16	GCTAGGTTGGATGAATCCTAC	GCGGCCGCCATCCAAAGTGTTACAAAG

miR-497	CCTGAGCTGAGTTCCTACAG	GCGGCCGCGTGTGGGCAACAAAGACTCC

miR-195	GTGACACAAAAAACATCTGG	GCGGCCGCATAAGCATCTGCCCCCAACC

miR-515-2	CGAGTAGCTGGGACTACAGG	GCGGCCGCATACCAGCTACTCGGGAGGC

miR-144	ATTACGCCATCTCTGGCTTG	GCGGCCGCACGTTTTCTGGGTATAGC

miR-34a	ATGGAGTCTTGCTAGTTGCC	GCGGCCGCTACGTGCAAACTTCTCCCAG

miR-449a	TTGAAGTAATACATTTTGTG	GCGGCCGCACTGTCTACTCCGAAAGAC

miR-146a	GTAGAGACAAATTCTCCATG	GCGGCCGCTTTCTCACAGGAACTCACAC

miR-655	AATCCGTGGAGGAAGCTTTC	GCGGCCGCACAGCAAACACAGCAAAGC

miR-374	GTTCCTTTATTGGAAGTCTG	GCGGCCGCAGGCACACAATAAATGTTTG

miR-524	AGTAGAGACAGGGTTTCACC	GCGGCCGCGATAGAGAGACTCTGTCCGC

miR-520d	CTGGTGTCTAACTCCTGAGA	GCGGCCGCTGACCATTTGAAGCCAAGAG

miR-376a-1	CCTGAGTAGGTGCAAAGATG	GCGGCCGCATTATGTGTGCACCAAGGGC

miR-23b	AATTGTGTGTGTCGCATGGC	GCGGCCGCGGTGCAGAACTTAGCCACTG

miR-23a	GCCATGCAAGTTGCTGTAGC	GCGGCCGCTTCCTGCTGAACTGAGCCAG

miR-199a-2	AGCGCATACCCAAACCTCCC	GCGGCCGCGTCAAGAAGGCCTTACCCGC

miR-199b	GGGGTTGGACACTAGGTAGG	GCGGCCGCAGGTTTTGGGGTGAGGGAGG

miR-194-1	CTAATGGCATTATCTCACAG	GCGGCCGCGATGACTTTAATCCTCTTCC

miR-202	CCTGCGCATAAATGTGGAGC	GCGGCCGCTCCTCTCTGCATCCTGGGTC

miR-875	TATACCTCAGTTTTATCAGGTG	GCGGCCGCGTGCATAGCTTCTGTAAAGG

miR-511-1	AATGGCCTCTAGTTGCATCC	GCGGCCGCATAGATCACTTACCTGAGGG

The luciferase-UTR reporter plasmids (Luc-CCND1-3'-UTR-1 and Luc-CCND1-3'-UTR-2) were constructed by introducing the CCND1 3'-UTR carrying putative microRNA binding sites into pGL3 control vector (Promega, Madison, WI). Thus, we amplified the CCND1 3'-UTR sequence from MCF10A cDNA by PCR using the primers as follows.

CCND1-UTR-5.2 (sense) 5'-GGGCGCCAGGCAGGCGGGCGC (1–21)CCND1-UTR-Not1-3.2 (antisense) 5'-**GCGGCCGC**TGGTTTTAGAATATGAAGAAG (981–1002) for Luc-CCND1-3'-UTR-1.

CCND1-UTR-5.3 (sense) 5'-ACGTCCAGGTTCAACCCACAG (944–965)CCND1-UTR-Not1-3.4 (antisense) 5'-**GCGGCCGC**GTCTTTTTGTCTTCTGCTGGA (3134–3155) for Luc-CCND1-3'-UTR-2. Using the same strategy, the amplified fragments were then cloned into the modified pGL3 where EcoR1 and Not1 sites were introduced so that the UTR sequences were unidirectionally cloned downstream the luciferase gene.

To delete miR-503 binding sites, we used a two step PCR procedure. In the first PCR reaction, 2 sets of primers CCND1-UTR-5.3 and CCND1-UTR-3.6 ACAAAAAACTGATCCTCCAAGCTGCGGCCTGTCCCCGGTGT; CCND1-UTR-5.6 ACACCGGGGACAGGCCGCAGCTTGGAGGATCAGTTTTTTGT (complementary to CCND1-UTR-3.6) and CCND1-UTR-Not1-3.4 were used to generate two overlapping PCR products where the miR-503 binding sites were deleted. In the second PCR reaction, we used these two products as templates and primers CCND1-UTR-5.3 and CCND1-UTR-Not1-3.4. Finally, the PCR product was cloned into the pGL3 control vector as above. All PCR products were verified by DNA sequencing.

### Transfection

UMSCC10B or 293T cells were transfected using DNAfectin reagent (Applied Biological Materials, British Columbia, Canada) following the manufacturer's protocol. In brief, cells were seeded at 40% confluence in a 12-well plate and then transfected with 1 μg of plasmid DNA in serum free medium the following day when the cells reached about 70% confluence. The serum free media was then replaced by normal growth medium after 16 h of transfection.

### Luciferase Assay

Luciferase assays were carried out in 293T cells to determine the effect of microRNAs on the activity of Luc-CCND1-3'-UTR and the deletion mutant constructs. First, cells were transfected with appropriate plasmids in 12-well plates. Then, the cells were harvested and lysed for luciferase assay 24 h after transfection. Luciferase activity was determined by using a luciferase assay kit (Promega) according to the manufacturer's protocol. Renilla luciferase was used for normalization.

### PCR/RT-PCR and real-time RT-PCR

PCR was performed to amplify pre-microRNA sequences or the CCND1 3'-UTR sequence according to the standard three-step procedure. Annealing temperature varied depending on the primers used. For RT-PCR, we isolated total RNA using Trizol reagent (Invitrogen) per the manufacturer protocol and used 1 μg RNA to synthesize cDNA by SuperScriptase III (Invitrogen) with random primers. Finally, the resultant cDNA was used in regular PCR or real-time PCR reactions. To detect CCND1 mRNA levels, we used the SYBR Green method with primers CCND1-5.1 and CCND1-3.1.

CCND1-5.1 (sense) 5'-CAGGAGAGGAAAGCATGGAG

CCND1-3.1 (antisense) 5'-TCGGGTGAAATAATGGTGGT

To detect mature microRNA expression, we also used Trizol reagent to isolate total RNA. Real-time PCR reactions were performed in ABI 7500 HT thermal cycler according to the manufacturer's protocol. Average levels of U6, 5s RNA and β-actin were used as an internal control. The fold-change between vector control and pre-microRNA expression vector was calculated with the 2^-ΔΔCt ^method [27,28].

### Cell growth inhibition assay

Cell growth assays were carried out by MTT [3-(4,5-Dimethylthiazol-2-yl)-2,5-diphenyltetrazolium bromide] as described previously [29]. In brief, cells were seeded in 96-well plates and incubated for various days before adding MTT. Absorbance at 570 nm was measured in the multi well plate reader (Thermo Scientific, Waltham, MA). The relative values were calculated by expressing the value at the first day as 1.

### Western Blot

Cells were harvested and protein was extracted 2 days after transfection as previously described [26]. Protein concentration was determined by protein assay kit (Bio-Rad, Hercules, CA) and samples were separated in 12% SDS polyacrylamide gels. Signals were revealed by a secondary antibody labeled with IRDye 800CW and the signal intensity was determined by Odyssey Infrared Imaging System (LI-COR Biosciences).

### Cell cycle analysis

Cell cycle analysis was performed using the standard propidium iodide method. In brief, UMSCC10B cells were first transfected with either vector control or miR-503 expression vector. One day later, cells were then split and grown for 24 h before harvesting for cell cycle analysis. After fixing at 70% ethanol, the cells were stained with propidium iodide along with RNase A. Finally, the cells were analyzed by FACS Vantage flow cytometer (Becton-Dickinson).

### Statistical analysis

Statistical analysis of data was performed using the Student's *t *test. Differences with p values less than 0.05 are considered significant.

## Results

**Figure 1:**
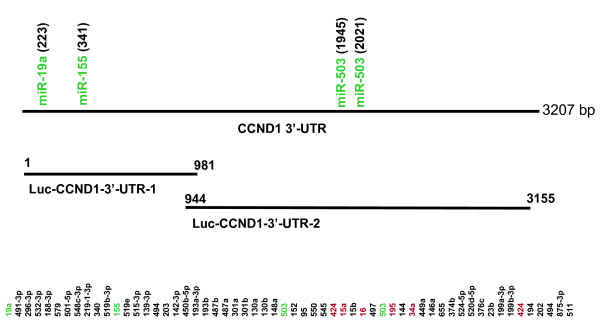
**The CCND1 3'-UTR with predicted microRNAs and luciferase constructs**. The CCND1 3'-UTR sequences (3207 bp) is based on NM_053056. Three microRNAs identified to be positive to CCND1 in this study are indicated in green with relative positions. Those previously validated microRNAs are indicated in red. See Table 2 for detail positions.

To determine microRNA regulation of CCND1, we searched for putative microRNAs that are able to target CCND1 based on commonly cited prediction programs such as miRanda [30]http://www.microrna.org and TargetScan4 http://www.targetscan.org[31], PicTar http://pictar.mdc-berlin.de/[32]. Apparently, different programs may predict different microRNAs for a given coding gene. We used miRanda as a primary source in this study. Based on January 2008 Release version, we found a total of 58 microRNAs that potentially target CCND1 (Fig. 1). To obtain a comprehensive picture of microRNA targeting for CCND1, we made an attempt to test all 58 predicted microRNAs.

**Figure 2:**
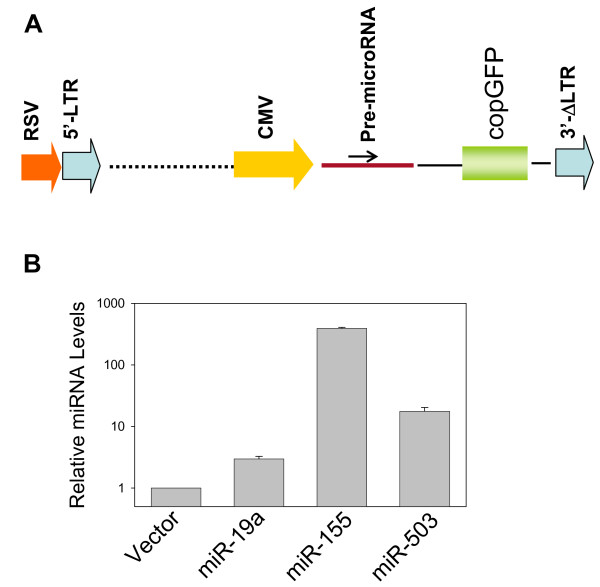
**Expression of exogenous microRNAs used for validation**. **A**, Schematic description of microRNA constructs. **B**, Expression of three microRNAs that were identified to be positive in this study. 293T cells were first transfected with indicated miRNAs and harvested 24 h after infection for extraction of RNA. The relative expression was determined by real-time PCR. Values are means of three transfection experiments.

**Figure 3:**
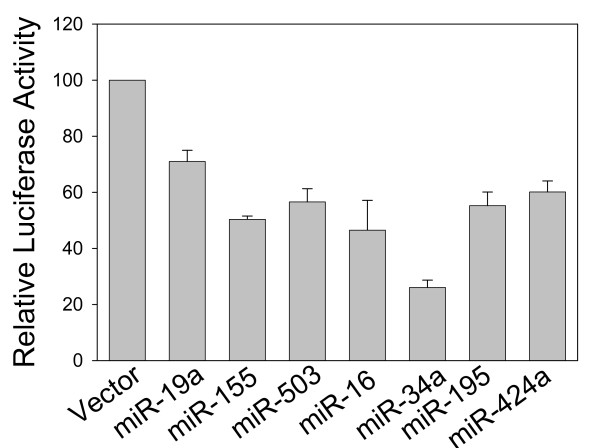
**Validation of seven microRNAs capable of silencing CCND1**. 293T cells were transfected with indicated Luc-CCND1-3'-UTR along with corresponding miRNAs and harvested 24 h after transfection for luciferase assays as detailed in Materials and Methods. Values are means ± SE of three transfection experiments. **, p < 0.01.

We first cloned all of these microRNA precursors into pCDH-CMV-MCS-EF1-copGFP (pCDH, System Biosciences, Mountain View, CA) expression vector. As indicated in Fig. 2A, the pre-microRNAs were expected to express under control of the CMV promoter and eventually processed into mature microRNA by the cellular Drasha and Dicer. Since this vector also carried green fluorescent protein (GFP) tag, transfection rate can be easily monitored under a fluorescence microscope. We were able to clone 51 of 58 microRNAs and then introduced into 293T cells. Two days after transfection, RNA was isolated, followed by real time RT-PCR analysis, which revealed that 45 of them gave rise to a higher level of mature microRNAs over the endogenous counterpart (vector control) (Table 2). On average, the exogenous microRNAs were up to a 100-fold higher than the endogenous microRNAs. The variations among different microRNAs are also in part due to variations in the levels of endogenous microRNAs. Therefore, the majority of these microRNAs were highly expressed. Shown in Fig. 2B were three microRNAs (miR-19a, miR-155 and miR-503) that were ectopically expressed because these three microRNAs were capable of suppressing Luc-CCND1-3'-UTR as demonstrated in this study (Fig. 3).

**Table 2:** Ectopic expression of microRNAs

Name	Vector	Vector IC	miR	miR IC	V:DCT	miR:DCT	DDCT	Fold change
19a	20.1	14.5	18.4	14.1	5.6	4.3	-1.2	2.4

491-3p	24.4	14.3	22.0	14.2	10.1	7.8	-2.4	5.1

296-3p	22.8	14.5	17.3	14.1	8.3	3.1	-5.2	36.1

532-3p	22.0	14.3	20.5	14.2	7.7	6.2	-1.5	2.9

188-3p	25.0	14.5	21.4	15.0	10.6	6.4	-4.2	17.9

579	27.3	14.5	23.3	14.1	12.9	9.2	-3.7	12.8

501-5p	23.0	14.5	19.2	14.1	8.5	5.1	-3.4	10.7

548c-3p	26.7	14.5	24.8	14.1	12.3	10.7	-1.6	3.1

219-1-3p	26.0	14.5	19.4	14.4	11.5	5.0	-6.5	92.9

519b-3p	28.6	14.3	22.9	14.2	14.3	8.7	-5.6	48.8

155	33.6	17.1	25.3	17.5	16.5	7.8	-8.7	415.9

519e	28.6	14.3	22.9	14.2	16.0	8.7	-7.3	155.0

139-3p	24.4	14.5	16.7	13.7	9.9	3.0	-6.9	118.5

203	26.2	14.5	15.3	14.1	11.7	1.2	-10.5	1436.2

142-3p	31.9	14.5	21.2	14.1	17.5	7.1	-10.3	1296.1

450b-5p	21.3	14.5	17.3	15.0	6.9	2.3	-4.5	23.2

193a-3p	24.9	14.5	19.0	14.4	10.4	4.7	-5.8	55.1

193b	24.0	14.3	20.3	14.1	9.7	6.2	-3.5	11.5

487b	26.3	14.3	17.6	14.1	12.1	3.4	-8.6	391.9

487a	28.3	14.5	19.0	15.0	13.9	4.1	-9.8	900.1

301a	25.1	14.5	22.0	15.0	10.6	7.1	-3.6	11.8

130a	25.0	14.3	16.1	14.1	10.8	2.0	-8.8	431.6

130b	20.4	14.5	17.4	14.3	5.9	3.2	-2.7	6.5

148a	21.1	14.7	16.6	14.6	6.4	2.0	-4.2	18.1

503	24.8	18.6	20.4	18.1	6.2	2.3	-3.9	14.5

152	24.2	14.5	15.5	14.1	9.7	1.4	-8.4	327.0

95	23.4	14.5	16.0	14.2	8.8	1.8	-7.0	128.3

550	21.7	14.5	16.1	14.4	7.2	1.7	-5.5	44.7

424	18.0	14.5	14.6	14.3	3.5	0.4	-3.1	8.5

16	16.8	14.3	15.6	14.2	2.5	1.4	-1.2	2.3

195	17.3	14.3	15.2	14.2	3.0	1.0	-2.1	4.2

144	30.1	14.5	24.2	14.2	15.6	10.0	-5.6	47.0

34a	27.4	15.5	19.0	14.6	11.9	4.4	-7.5	187.3

449a	25.2	14.3	15.6	14.2	10.9	1.4	-9.5	723.8

146a	34.6	14.5	25.6	14.5	20.1	11.1	-9.0	505.0

655	29.7	14.5	27.4	14.2	15.2	13.3	-1.9	3.8

374b	19.5	14.5	16.8	14.2	5.0	2.7	-2.3	5.1

520d-5p	31.8	14.5	25.8	13.7	17.3	12.1	-5.2	36.8

376c	29.3	14.5	18.7	14.1	14.9	4.6	-10.3	1296.1

23b	19.2	14.3	18.0	14.2	4.9	3.7	-1.2	2.2

199a-3p	27.6	14.5	17.5	14.1	13.1	3.4	-9.8	864.1

194	23.7	14.5	17.4	14.1	9.2	3.2	-6.0	62.4

202	31.2	14.5	18.4	13.7	16.7	4.7	-12.0	3959.2

875	23.4	14.5	15.7	13.6	8.8	2.1	-6.7	104.5

511	25.4	14.5	20.5	14.4	10.9	6.1	-4.9	29.1

Values are C_T_s except for fold change. IC, Internal controls, average of 5sRNA, U6 and β-actin; V, vector.

Next, we cloned the CCND1 3'-UTR (NM_053056). As indicated in Fig. 1, the CCND1 3'-UTR was about 3207 bp in length with corresponding microRNA sites. Since this fragment was relatively long, we divided it into two fragments and separately cloned into pGL3 control vector, generating Luc-CCND1-3'-UTR-1 and Luc-CCND1-3'-UTR-2, which were overlapped by 58 bps. Having demonstrated ectopic expression of these microRNAs and construction of CCND1-3'-UTR luciferease reporters, we introduced each of microRNAs listed in Table 2 into 293T cells along with Luc-CCND1-3'-UTR-1 or Luc-CCND1-3'-UTR-2. Luciferase assays indicated that although the majority of microRNAs had no effect on its luciferase activity, a total of seven microRNAs, miR-19a, miR-153, miR-503, miR-16, miR-34a, miR-195 and miR-424a suppressed its luciferase activity by over 35% (Fig. 3). Among those microRNAs, miR-16, miR-34a, miR-195 and miR-424a were previously reported to suppress CCND1 [22,23,33]. On the other hand, miR-19a, miR-155, and miR-503 have not been previously validated to target CCND1. Therefore, we further characterized these three microRNAs.

**Figure 4:**
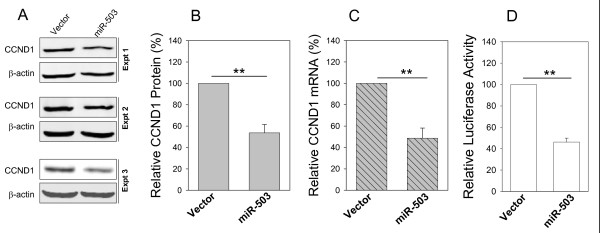
**Suppression of the endogenous CCND1 by miR-503**. **A **and **B**, Western blot revealing reduction of CCND1 protein in miR-503 transfected UMSCC10B cells with quantitative measurement of three experiments (**B**). **C**, The same miR-503 transfected UMSCC10B cells were subject to RNA extraction and real time PCR analysis. D. Luciferase assays in UMSCC10B cells. Values in B, C and D are means ± SE of three separate experiments. **, p < 0.01. Expt, Experiment.

To further demonstrate that these microRNAs truly target CCND1, we determined whether they are able to suppress the endogenous CCND1 protein levels. Western blot analysis indicated that only miR-503 was able to suppress CCND1 protein by over 40% (Fig. 4A and 4B) in UMSCC10B cells in addition to its ability to suppress the luciferase activity (Fig. 4D). Moreover, we examined the CCND1 mRNA by real time PCR and found that only miR-503 significantly reduced CCND1 mRNA (Fig. 4C). These results suggest that miR-503 is a true microRNA for CCND1 whereas it remains to be determined for their targeting capability for the other two microRNAs.

**Figure 5:**
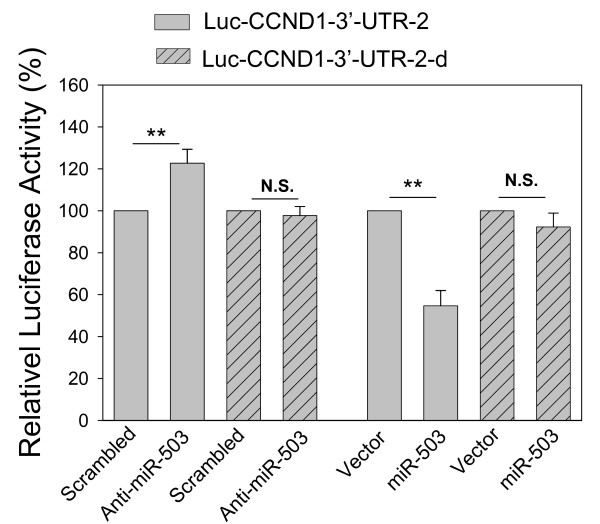
**Effect of anti-miR-503 and miR-503 binding sites on the Luc-CCND1-3'-UTR activity**. 293T cells were transfected with Luc-CCND1-3'-UTR-2 along with either scrambled oligo or anti-miR-503 and the harvested for luciferase assays 24 h after transfection. To determine the importance of miR-503 binding sites, the same cells were transfected with Luc-CCND1-3'-UTR-2-d, in which the miR-503 binding sites were deleted. Along with the reporter Luc-CCND1-3'-UTR-2-d was either vector or miR-503. Values are means ± SE of three separate experiments. **, p < 0.01; n.s., not significant.

To confirm that suppression of CCND1 is specific to miR-503, we transfected the cells with anti-miR-503 along with the reporter. In contrast to miR-503, anti-miR-503 caused an increase in the luciferase activity compared to the scrambled oligo (Fig. 5). To determine the importance of two miR-503 binding sites, we deleted these two sites. This deletion substantially impaired its suppression activity, suggesting that these two putative bindings are important for miR-503-mediated suppression.

**Figure 6:**
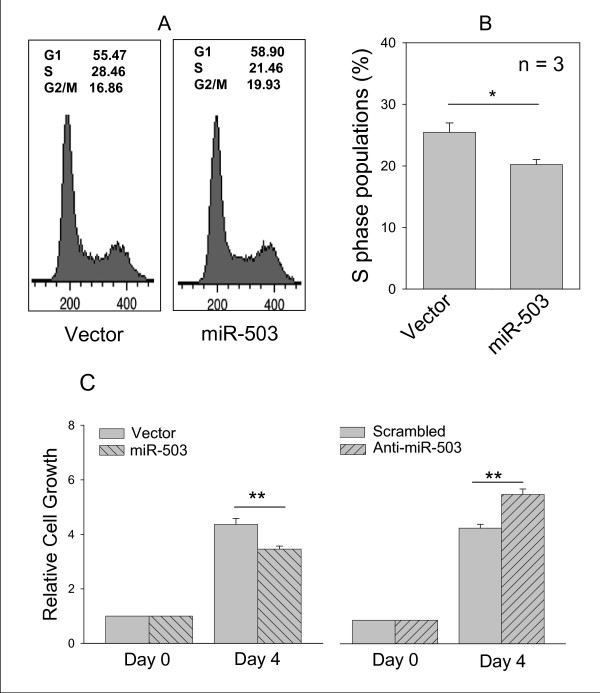
**Effect of miR-503 on cell cycle and cell growth**. **A**, A representative cell cycle profile for vector control or miR-503 in UMSCC10B cells. **B**, Average of S phase populations. **C**, While miR-503 suppresses, anti-miR-503 increases cell growth. UMSCC10B cells were transfected with vector or miR-503, or scrambled oligo or anti-miR-503 and then grown for 4 days before MTT assays. Values in **C **are means ± SE of three separate experiments. **, p < 0.01; *, p < 0.05.

Given that CCND1 is a key protein regulating cell cycle, we then determined whether suppression of CCND1 by miR-503 has any effect on cell cycle. We found that miR-503 slightly increased G1 phase populations, but had more profound effect on S phase populations (Fig. 6A). Based on average of three separate experiments, suppression of S phase populations by miR-503 was statistically significant (Fig. 6B). While the vector control revealed 25% of S phase populations, miR-503 cells revealed 20% of S1 phase populations. This result is consistent with the previous report that higher levels of CCND1 are associated with higher S phase populations in cancer cells [34].

Suppression of CCND1 by miR-503 is expected to reduce cell growth. Therefore, we determined the effect of miR-503 on cell growth by MTT assays. As shown in Fig. 6C, miR-503 suppressed cell growth by about 31% compared to vector control. On the other hand, anti-miR-503 enhanced cell growth by about 30%.

## Discussion

Although there are numerous computer-aided algorithms for prediction of microRNA targets, these predicted targets remains to be experimentally validated and how many microRNAs can actually target a single gene is not clear. Our study suggests that the ratio of true target/predicted target is relatively low in our system, and further suggest the complexity of the microRNA/mRNA interactions. There are numerous putative target genes for a given microRNA. Similarly, for given protein coding gene, there are often many putative microRNAs. Therefore, it would be of merit to determine how many microRNAs can actually target a given gene. We chose CCND1 for two reasons. First, it is a well known proto-oncogene that is implicated in tumorigenesis. Second, CCND1 has a relative long UTR so that a relative large number of putative microRNAs can target it, which would allow us to make a good assessment of microRNA targeting.

Among the 58 predicted microRNAs, we cloned 51 of them. We found that 6 microRNA precursors did not express sufficient amount of exogenous microRNAs. Therefore, these microRNAs were excluded from validation. Among 45 microRNAs tested there were seven microRNAs found to be positive based on luciferase assays by arbitrary cutoff of 35% reduction. Thus, the positive ratio for tested microRNAs is about 16%. Although some other microRNAs such as let-7b and miR-17 were showed to be positive to CCND1 by other groups [21,24], these microRNAs are not in the miRanda list and thus, they were not tested in this study. As listed in Table 3, the predicted microRNAs can be categorized into 4 groups. The first group consists of 6 microRNAs which are overlapped in all three programs. Four of them have been validated in the literature or in this study. The second group consisting of microRNAs overlapped within miRanda and PicTar; this group has an additional microRNA, i.e., miR-195, which is also positive to CCND1. The third group of microRNAs are overlapped within miRanda and TargetScan4, consisting of 27 microRNAs. Among them, we have validated miR-503 and miR-19a positive to CCND1 by luciferase assays. The fourth group shares no overlapped microRNAs with any of other programs. However, we still found one potential microRNA (miR-424) for CCND1. Based on these comparisons, there appears to be a tendency that the first group gives rise to the highest possibility of positivity and the last group gives rise to the lowest possibility of positivity. However, there is a possibility that functional microRNA might be found in this group, highlighting the necessity of experimental validation.

**Table 3:** Comparison of predicted CCND1 microRNAs

Group 1 (M, T and P)						
**miR-15a**	**miR-15b**	miR-16	miR-34a	miR-23a	miR-23b	

**Group 2 (M and P)**						

**miR-15a**	**miR-15b**	miR-16	miR-34a	miR-195	miR-23a	miR-23b

**Group 3 (M and T)**						

**miR-15a**	**miR-15b**	miR-16	miR-34a	miR-195	miR-424	*miR-503*

*miR-19a*	miR-23a	miR-23b	miR-202	miR-497	miR-193a	miR-193b

miR-449a	miR-511	miR-494	miR-487a	miR-655	miR-95	miR-374b

miR-494	miR-296-3p	miR-548c-3p	miR-548d	miR-548c	miR-520d-5p	

**Group 4 (M only)**						

*miR-155*	miR-491-3p	miR-296-3p	miR-532-3p	miR-188-3p	miR-579	miR-501-5p

miR-219-1-3p	miR-340	miR-519b-3p	miR-519e	miR-515-3p	miR-139-3p	miR-203

miR-142-3p	miR-450-5p	miR-487b	miR-301a	miR-301b	miR-130a	miR-130b

miR-148a	miR-152	miR-550	miR-545	miR-497	miR-144	miR-449a

miR-146a	miR-655	miR-374b	miR-524-5p	miR-520d-5p	miR-376c	miR-199a-3p

miR-199b-3p	miR-194	miR-202	miR-875-3p	miR-511		

*M, miRanda; T, TargetScan4; P, PicTar. microRNAs in bold only were validated in literature; those underlined were validated in both literature and in this study. Those in *italicized* were validated in this study only.

Given that CCND1 can be targeted by multiple microRNAs, it is our expectation that those microRNAs may simultaneously target CCND1 in the same cell in concert if they are present at the same time. In this case, it would be interesting to determine whether they have a synergistic suppressive effect on CCND1. In addition to the previously identified microRNAs such as miR-16 and miR-34a, we found that microRNAs such as microRNA-503, which have not been previously reported, can also target this gene. Four lines of evidence support that miR-503 specifically targets CCND1. First, ectopic expression of miR-503 suppresses luciferase assays with Luc-CCND1-UTR. Second, anti-miR-503 enhances the activity. Third, miR-503 does not only affect the luciferase activity, but also the endogenous gene, as shown by western blot and real time PCR. Forth, miR-503 suppresses tumor cell growth, consistent with the notion that suppression of CCND1 would inhibit cell growth. Although there is little information available in the literature regarding function of miR-503, our findings suggest that miR-503 is a putative tumor suppressor.

The other two potential microRNAs (miR-19a and miR-155) are also interesting because they show suppression ability for the luciferase activity. However, they do not seem to affect CCND1 protein or mRNA levels, which could be due to the cell type specific effect or artifacts that might be caused by luciferase assays. This remains to be further determined.

In summary, we used a luciferase reporter system and systematically tested miRNAs that are predicted to target CCND1 by a computer-aided prediction method. Our validation results provide a relatively comprehensive picture of miRNA/mRNA interactions. Thus, this information may be valuable for future studies on the regulation of this important gene.

## Conclusion

This study suggests that miRNAs can interact with a target mRNA at both conserved and non-conserved sites although the conserved sites give rise to a higher positive ratio than the latter. The overall ratio for positive target sites among the predicted sites is relatively low. However, since it is possible that miRNA/mRNA interactions may depend on the cellular content, this ratio could be different in a different cellular content. Therefore, our study highlights the importance of experimental target validation.

## Abbreviations

PCR: polymerase chain reaction; RT: reverse transcription; UTR: untranslated region.

## Competing interests

The authors declare that they have no competing interests.

## Authors' contributions

QJ, MF and YM designed research, analyzed data and wrote the paper; QJ performed experiments. All authors read and approved the final manuscript.

## Pre-publication history

The pre-publication history for this paper can be accessed here:

http://www.biomedcentral.com/1471-2407/9/194/prepub
